# A Comparative Study on Microstructure and Mechanical Properties of Friction Stir Welded Butt Joints Produced Under Different Cooling Conditions in a High-Strength Al–Cu–Li Alloy and an Al–Zn–Cu–Mg Alloy Modified with Ag, Zr, and Sc

**DOI:** 10.3390/ma19214578

**Published:** 2026-10-29

**Authors:** S. Tarasov, E. Sidorov, D. Gurianov, V. Rubtsov, S. Fortuna, K. Osipovich, E. Kolubaev, A. Chumaevskii

**Affiliations:** Institute of Strength Physics and Materials Science, Siberian Branch of Russian Academy of Sciences, Tomsk 634055, Russia

**Keywords:** aluminum alloys, friction stir welding, cooling conditions, precipitates, strength

## Abstract

**Highlights:**

- Synergistic mechanisms between advanced microalloying elements and external cooling rates during FSW were established.
- Two fundamentally different precipitation-hardening systems were revealed.
- A reliable pathway to engineer high-performance FSW joints is offered.

**Abstract:**

This study provides a comprehensive and novel comparative investigation into the evolution of microstructure and mechanical properties of FSW butt joints in a high-strength Al–Cu–Li alloy and an Al–Zn–Cu–Mg alloy modified with Ag, Zr, and Sc, processed under different cooling conditions (ambient air and forced water cooling). Mechanical performance was evaluated via transverse tensile testing and microhardness profiling. The scientific novelty of this work lies in revealing the distinct phase-stability and grain-refinement mechanisms governing each alloy under altered thermal cycles. The experimental results reveal that forced water cooling effectively restricts the peak welding temperature and curtails thermal exposure time, thereby significantly suppressing dynamic recrystallization grain growth in the stir zone (SZ) for both alloys. For the first time, a critical divergence in the post-weld hardening response was established: in the Al–Cu–Li alloy, accelerated cooling partially prevents the complete dissolution of the metastable T1-Al_2_CuLi phase, driving higher joint recovery. Conversely, the Ag-, Zr-, and Sc-modified Al–Zn–Cu–Mg alloy demonstrates superior structural stability; the thermally stable Al_3_(Sc, Zr) nanoprecipitates were found to exert a powerful Zener pinning effect, maintaining an ultra-fine grain structure (≤1 µm) even under ambient air conditions and completely preventing grain boundary shearing during rapid cooling. Consequently, the water-cooled modified Al–Zn–Cu–Mg joints achieved an exceptional joint efficiency of over 85%, shifting the typical HAZ failure location toward the thermomechanically affected zone (TMAZ) boundary.

## 1. Introduction

Al–Cu–Li and Al–Zn–Mg–Cu (modified with Ag, Zr, Sc) aluminum alloys are widely utilized in aerospace and rocket engineering to achieve weight reduction while maintaining high specific strength and stiffness. The development of third-generation alloys, including AA2050, AA2195, AA2198, AA2098, and AA2199, is driven by the necessity to combine superior strength, processability, corrosion resistance, and cyclic fatigue life [1,2,3]. The AA2050 alloy, specifically developed for medium- and thick-gauge sheets and plates, represents a typical example of this group. The high mechanical properties of Al–Cu–Li alloys are governed by the state of the solid solution and secondary dispersed phases, primarily T_1_-Al_2_CuLi, θ′-Al_2_Cu and δ′-Al_3_Li. The precipitation of these phases depends on the chemical composition, pre-deformation, quenching conditions, and artificial aging regimes.

The properties of high-strength Al–Zn–Cu–Mg alloys are also dictated by precipitation hardening mechanisms. Following quenching and aging, solute clusters, Guinier–Preston (GP) zones, and metastable η′-MgZn_2_ form within the matrix. Although increasing the Zn, Mg, and Cu content enhances strength, it simultaneously narrows the allowable temperature window for heat treatment. During conventional fusion welding, such alloys are highly prone to hot cracking, element segregation, and the formation of a coarse as-cast structure. Micro-alloying with scandium extends the possibilities for microstructural control. In the presence of Zr, scandium forms coherent Al_3_(Sc, Zr) dispersoids that remain stable at hot deformation and welding temperatures. These dispersoids effectively pin grain boundaries via the Zener mechanism and suppress grain growth during recrystallization. The influence of Sc and Zr on the structure of Al–Zn–Mg–Cu alloys has been extensively studied for aged, deformed, and spray-formed materials [4,5,6,7,8].

Friction stir welding (FSW) enables the joining of these advanced alloys without melting the base metal. A rotating tool heats the material to a plasticized state, stirs it, and forms the weld joint through intense plastic deformation and frictional heating. Three distinct zones are typically identified in the welded joint: the stir zone or weld nugget (SZ), the thermo-mechanically affected zone (TMAZ), and the heat-affected zone (HAZ). These regions differ significantly in grain size and morphology, dislocation density, texture, residual stress levels, and the state of the strengthening precipitates. For the AA2199 alloy, microstructural mapping has allowed researchers to track structural evolution across the weld cross-section and correlate local properties with the varied thermo-mechanical history of the material. Similar trends have been identified for AA2050 in the T8 temper [9] and for thin-sheet Al–Cu–Li alloys using transmission electron microscopy [10,11].

For the AA2195 alloy after FSW, modifications of precipitate morphology and weld joint structure have been established, driven by the combined effects of frictional heating, superplastic flow, dynamic recrystallization, and dynamic precipitation. Using the example of an FSWed AA2198 alloy, it was demonstrated that the fine-grained SZ microstructure contributes to strengthening according to the Hall–Petch relation [12,13] and dispersion hardening, whereas internal weld defects may noticeably degrade joint strength. For AA2060, the microstructural evolution and mechanical properties resulting from FSW have been described to confirm that the distribution of structural states across the weld cross-section depends both on the FSW thermo-mechanical conditions and the post-weld heat treatment parameters [14,15,16,17]. Consequently, the direct transfer of FSW parameters between different Al–Cu–Li alloys without additional experimental validation is fundamentally incorrect.

The relationship between the FSW thermal cycle and subsequent heat treatment has been demonstrated for Al–Cu–Li alloys by several authors. Sidhar and Mishra [18] investigated the aging kinetics of friction stir welded joints in Al–Cu–Li–Mg–Ag and Al–Cu–Li–Mg systems. For AA2050, it was established that controlling the thermal conditions during welding alters the kinetics of subsequent artificial aging. Studies [15,16,17] also showed that the post-weld microstructure can significantly evolve during additional heating. These data confirm that forced cooling and subsequent artificial aging should be treated as interrelated technological stages.

When applied to AA7075-T651 and AA7050 alloys, FSW reduces the probability of defect formation characteristic of fusion welding; however, the weld zone structure remains highly heterogeneous [19,20,21,22,23,24]. The TMAZ and HAZ experience different thermal impacts, involving particle coarsening, element partitioning, and the formation of precipitate-free zones (PFZs). Welding models for 7xxx alloys and material flow studies in AA7075 correlate these processes with the tool rotational speed, welding speed, pin geometry, and heat dissipation conditions [25,26,27]. Similar dependencies have been established for AA7075-O, AA7050, and thick-plate Al–Zn–Mg–Cu alloys [28,29,30].

The “weakening” of the HAZ remains the primary metallurgical challenge during the FSW of high-strength Al–Zn–Mg–Cu alloys. The peak temperature in this zone is sufficient to dissolve or coarsen the strengthening phases but insufficient for recrystallization to occur. Consequently, a region with a minimum microhardness is formed (exhibiting a W-type microhardness profile), which dictates the localization of deformation and subsequent tensile failure. In situ measurements and modeling performed by dos Santos et al. demonstrated the direct link between the welding thermal cycle and the evolution of the η′- and δ′-phases [31]. The influence of welding parameters on the microstructure and strength has also been described for high-strength Al–Zn–Mg–Cu, AA7A04, and AA7A52 alloys [32,33,34,35].

This problem is even more pronounced in FSW performed on thick aluminum alloy sheets. Temperature history and cooling rates vary via a steep gradient across the plate thickness; thus, the structures formed beneath the weld surface, in the center of the stir zone, and near the weld root differ substantially. Fatigue tests of AA7050 and Al–Zn–Mg–Cu joints confirm that the formation of local low-hardness zones facilitates both crack initiation and propagation kinetics [36,37,38]. To address this issue, researchers utilize underwater welding, forced liquid cooling, specific controlled heat input schemes, and modified FSW regimes [39,40,41,42,43].

Cooling during the welding process allows for effective management of the thermal cycle without altering the base metal composition. Under air-cooling conditions, the heat dissipation intensity is mainly limited by the low thermal conductivity of the workpiece and the backing plate. Liquid cooling lowers peak temperatures and shortens the dwell time of the metal within the temperature interval favorable for the dissolution and coarsening of strengthening phases. During the underwater welding of the 7055 alloy, a more finely dispersed state of Mg–Zn-containing particles was noted compared to joints produced under standard conditions. Investigations of Al–Zn–Mg–Cu joints under intensified cooling also correlate the heat dissipation rate with the final grain size, phase composition, and microhardness profile [41,42,43,44].

The FSWed material is characterized by a thermodynamically non-equilibrium state. The solid solution composition, dislocation density, and nucleation site density for excess phases vary across the joint zones. Subsequent artificial aging enables partial recovery of the strength properties, but its efficiency is predetermined by the structure formed directly during welding and cooling. This is critically important for joints produced using liquid cooling, as accelerated heat dissipation alters not only the grain size but also the initial conditions for the subsequent decomposition of the supersaturated solid solution. Studies on the natural and artificial aging of Al–Zn–Mg–Cu welded joints have shown a significant impact of the post-weld heat treatment on hardness, strength, and corrosion resistance [45]. The state of grain boundary precipitates and the width of precipitate-free zones (PFZs) directly determine the susceptibility of such joints to stress corrosion cracking and intergranular failure.

For Sc-modified Al–Zn–Mg–Cu alloys, these insights, including detailed numerical thermal modeling data, have been previously reported [46,47,48,49]. In turn, microalloying with silver (Ag) exerts a profound influence on the kinetics of precipitation hardening by accelerating the nucleation and promoting the structural refinement of the η′-phase precipitates and enhancing the thermal stability of the structure.

Although a substantial body of literature on the friction stir welding of various Al–Zn–Mg–Cu alloys has accumulated, the specific impact of altered cooling rates on the joint performance of advanced Ag-, Zr-, and Sc-modified Al–Zn–Mg–Cu systems has been explored to a limited extent. Crucially, it remains unclear how ambient air versus forced liquid cooling pathways alter the microstructural evolution and transient phase states across the distinct weld zones under the concurrent presence of these multiple microadditives. To address this knowledge gap, the present work aims to provide a comprehensive comparative analysis of the microstructure evolution, phase composition, and mechanical properties of FSW joints fabricated from promising Al–Cu–Li and Al–Zn–Mg–Cu (Ag, Zr, Sc) aluminum alloys subjected to various localized heat dissipation regimes and subsequent post-weld artificial aging.

## 2. Materials and Methods

The microstructure, phase composition, and mechanical properties of friction stir welded (FSW) butt joints were systematically investigated using high-strength Al–Cu–Li and Ag/Zr/Sc-modified Al–Zn–Mg–Cu aluminum alloy sheets with a thickness of 1.9–2.0 mm. Welding was performed using a tool configuration featuring a 1.8 mm pin height and a 12 mm shoulder diameter. The pin was plunged along the butt joint line with zero clearance to a depth of 0.1–0.2 mm less than the sheet thickness. To ensure operational safety and prevent mechanical contact during tool rotation, the clearance between the modular coolant hose nozzle and the rotating tool was minimized to the lowest practically allowable limit.

**Figure 1:**
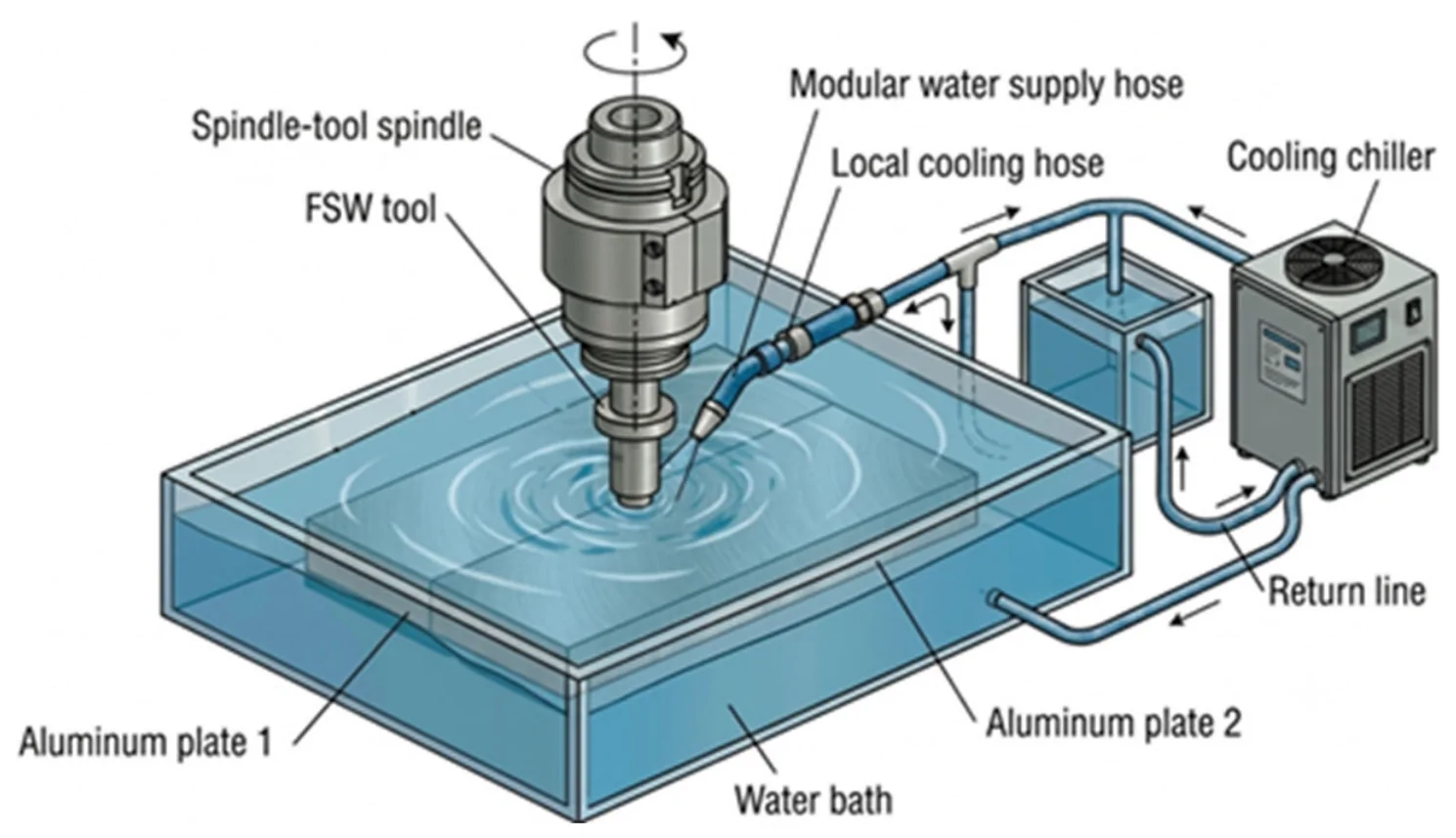
Schematic representation of the cooling medium delivery system for friction stir welding joint formation.

To evaluate the direct impact of localized heat dissipation and post-weld thermal history, specimens were extracted via electrical discharge machining (EDM) and tested in both the as-welded and post-weld artificially aged conditions. The butt joints were fabricated using the specialized FSW laboratory equipment at the Institute of Strength Physics and Materials Science of the Siberian Branch of the Russian Academy of Sciences (ISPMS SB RAS). For the controlled thermal management experiments, this setup was retrofitted with an external liquid-cooling system, comprising a specialized water bath, a cooling chiller, and a modular flexible hose assembly integrated with a precision flow meter (Figure 1).

Prior to the welding process, the cooling chiller was activated to reduce the water temperature from ambient room conditions down to 8–12 °C. Subsequently, water was supplied directly to the workpieces (which were rigidly secured to the worktable using mechanical clamping plates) immediately behind the trailing edge of the FSW tool at the onset of welding, concurrently filling the specialized cooling bath. The welding regime parameters are displayed in Table 1.

Tensile test specimens were extracted via electrical discharge machining such that the stir zone was precisely positioned at the geometric center of the gauge section, with the tensile axis oriented perpendicular to the joint line. To evaluate the post-weld precipitation kinetics, one batch of specimens was tested in the as-welded condition, while the remaining batch was subjected to post-weld artificial aging in a muffle furnace. The artificial aging regimes were set to 170 °C for 32 h for the Al–Cu–Li FSW joints, and 160 °C for 36 h for the Ag/Zr/Sc-modified Al–Zn–Mg–Cu FSW joints.

Microhardness profiling across the weld cross-sections was performed using a TBM 5215 A tester (Tochpribor, Ivanovo, Russia) equipped with a Vickers indenter, strictly in accordance with the ASTM E384 standard [50]. A wiping dwell time of 10 s and a test load of 50 g (0.49 N) were applied. The measurements were initially recorded on the Vickers hardness scale (HV) and subsequently converted into gigapascals (GPa) to facilitate thermodynamic and mechanical correlations.

## 3. Results

### 3.1. Macro- and Microstructure of FSW Joints on the Al–Cu–Li Alloy

**Figure 2:**
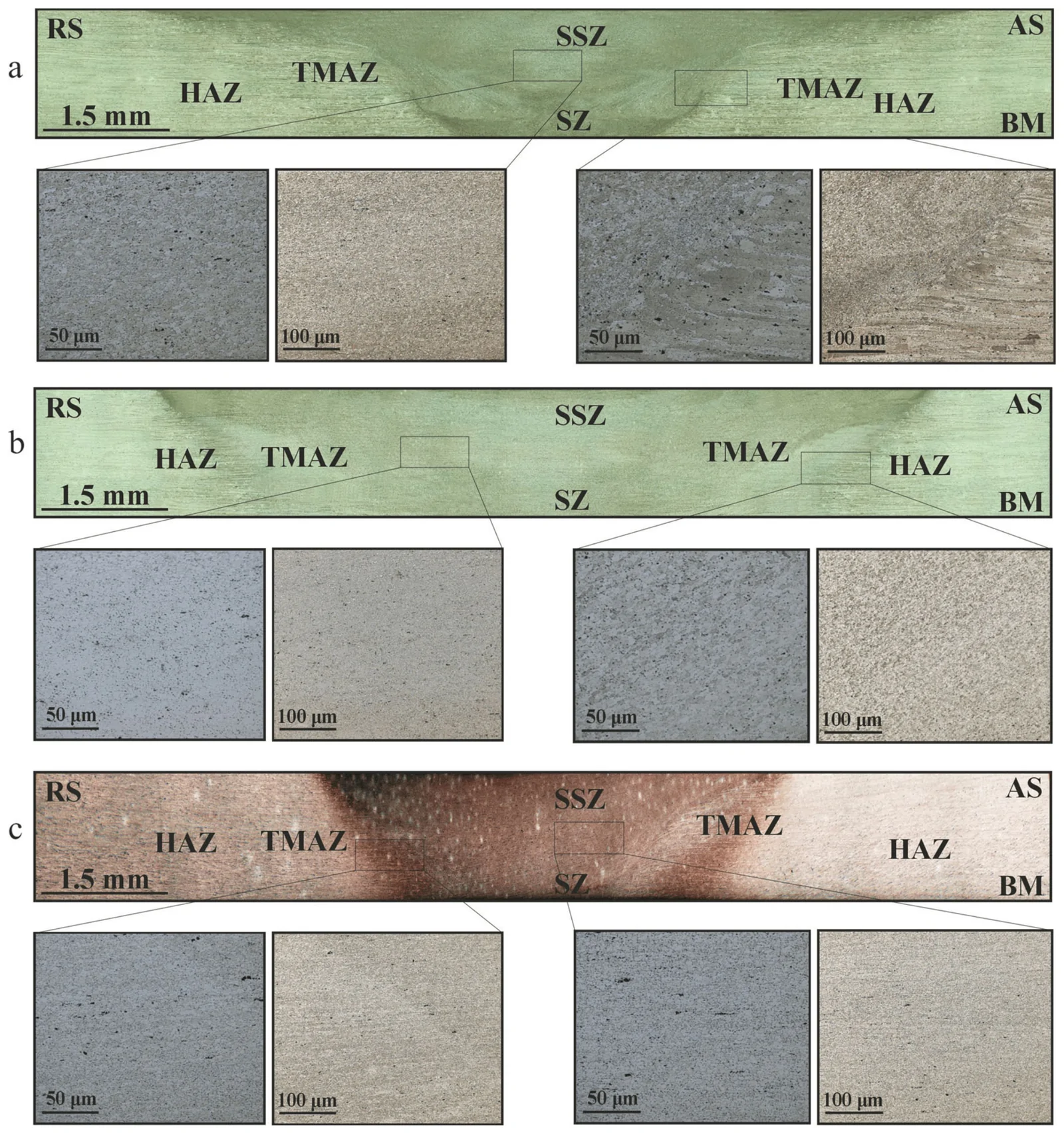
Macro- and microstructure of the FSW joint on the Al–Cu-Li alloy: (**a**) air cooling, (**b**) liquid cooling, (**c**) liquid cooling followed by artificial aging.

The panoramic macrosections of all investigated Al–Cu–Li alloy joints reveal a zonal structure characteristic of friction stir welding, which comprises the base metal (BM), heat-affected zone (HAZ), thermo-mechanically affected zone (TMAZ), stir zone (SZ), and shoulder-affected stir zone (SSZ) (Figure 2a–c).

In the joint processed under ambient air-cooling conditions, the stir zone (SZ) and the adjacent thermo-mechanically affected zone (TMAZ) differ noticeably from the base metal in terms of their etching contrast and localized structural heterogeneity. The optical micrographs exhibit individual dark spots corresponding to micro-voids left by pulled-out secondary particles, alongside regions characterized by varying etching intensities. For Al–Cu–Li alloys, this behavior is expected, as FSW radically alters the dislocation density, dissolves metastable strengthening precipitates, induces their coarsening or spatial redistribution, and modifies the misorientation state of grain and subgrain boundaries [9,10,11,15,17,20,51]. Prior investigations on AA2195, AA2198, and AA2050 welded joints have demonstrated that the most significant restructuring of the strengthening phases typically occurs within the SZ and TMAZ, where the coupled effect of elevated temperature and intense plastic deformation (thermomechanical coupling) is most pronounced [15,17].

Under forced liquid-cooling conditions, the typical sequence of weld zones is preserved, and the corresponding micrographs reveal excellent microstructural continuity without any visible macro-scale defects. Under the experimental conditions of this study, no obvious structural flaws were observed in the polished cross-sections. The accelerated heat dissipation effectively restricts the dwell time of the material within the critical temperature interval favorable for the dissolution and coarsening of strengthening nanoprecipitates. Consequently, liquid cooling alters the dynamic recrystallization kinetics, stabilizes the highly dispersed state of secondary phases, and mitigates the severe thermal degradation of the matrix inherent to conventional FSW [52,53,54,55]. Comparable conclusions have been reached in previous investigations regarding the role of controlled heat dissipation through the backing plate, optimized tool pin geometries, and active cooling regimes during the solid-state joining of lithium-bearing aluminum alloys.

Following forced liquid cooling combined with subsequent post-weld artificial aging, the panoramic structure maintains its continuity. Concurrently, post-weld aging alters the structural state of the material throughout the entire thermomechanically affected region. In Al–Cu–Li alloys, the enhancement of mechanical properties after artificial aging is traditionally attributed to the homogeneous nucleation or partial recovery of highly dispersed T_1_-(Al_2_CuLi), θ-(Al_2_Cu) and δ-(Al_3_Li), precipitates, as well as their direct interaction with mobile dislocations and the subgrain boundaries. The specific regions where partial dissolution or coarsening of the initial reinforcing particles occurred during the welding thermal cycle exhibit the highest sensitivity to such heat treatment; thus, the local precipitation hardening response within the SZ, TMAZ, and HAZ varies substantially [9,51,56].

### 3.2. Macro- and Microstructure of FSW Joints on the Al–Zn–Cu–Mg (Ag, Zr, Sc) Alloy

**Figure 3:**
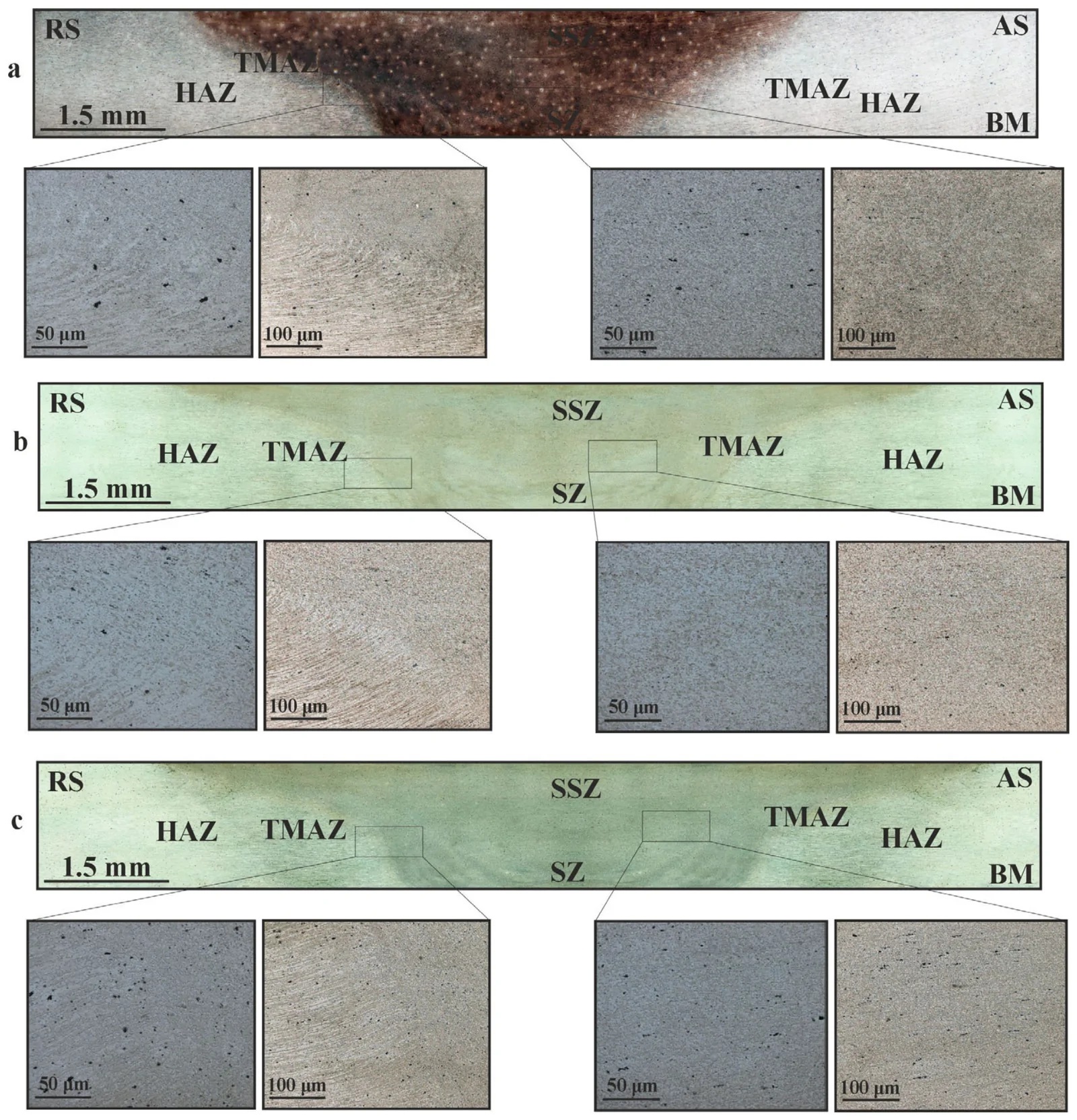
Macro- and microstructure of the FSW joint on the Al–Zn–Cu–Mg (Ag, Zr, Sc) alloy: (**a**) air cooling, (**b**) liquid cooling, (**c**) liquid cooling followed by artificial aging.

In the Al–Zn–Cu–Mg (Ag, Zr, Sc) alloy joint processed by FSW under ambient air-cooling conditions (Figure 3a), the stir zone (SZ) is clearly delineated. The SZ morphology exhibits a characteristic asymmetry relative to the retreating side (RS) and the advancing side (AS), with the transition boundary toward the TMAZ differing in width between both sides due to the nature of the thermomechanical material flow. Near the weld centerline, the material undergoes more intensive stirring, whereas the TMAZ structurally retains elongated grain features inherited from the initial cold-deformed base metal matrix.

Following forced liquid cooling (Figure 3b), the transition boundaries between the characteristic weld zones appear less distinct, and the overall joint structure demonstrates enhanced macroscopic homogeneity. Under the experimental conditions investigated, the stir zone maintains complete microstructural continuity, exhibiting no visible internal macro-defects or discontinuities in the polished cross-sections. The optical micrographs reveal a highly refined grain structure within the SZ, and the structural transition across the adjacent thermo-mechanically affected regions occurs gradually.

Post-weld artificial aging of the liquid-cooled joint (Figure 3c) does not induce any noticeable macrostructural rearrangement. The geometric profile of the SZ and the spatial boundaries of the TMAZ and HAZ remain virtually identical to those observed in the as-welded liquid-cooled state. Consequently, the primary metallurgical impact of post-weld aging is associated not with geometric alterations of the weld zones, but with the nanoscale nucleation and redistribution of strengthening precipitates within the aluminum matrix. While these sub-micron phase evolutions remain beyond the resolution limits of optical microscopy, their definitive influence is directly reflected in the localized microhardness profiles, macroscopic tensile properties, and high-resolution electron microscopy data discussed below.

### 3.3. Fine SZ Structure in an FSWed Al-Cu-Li Alloy

**Figure 4:**
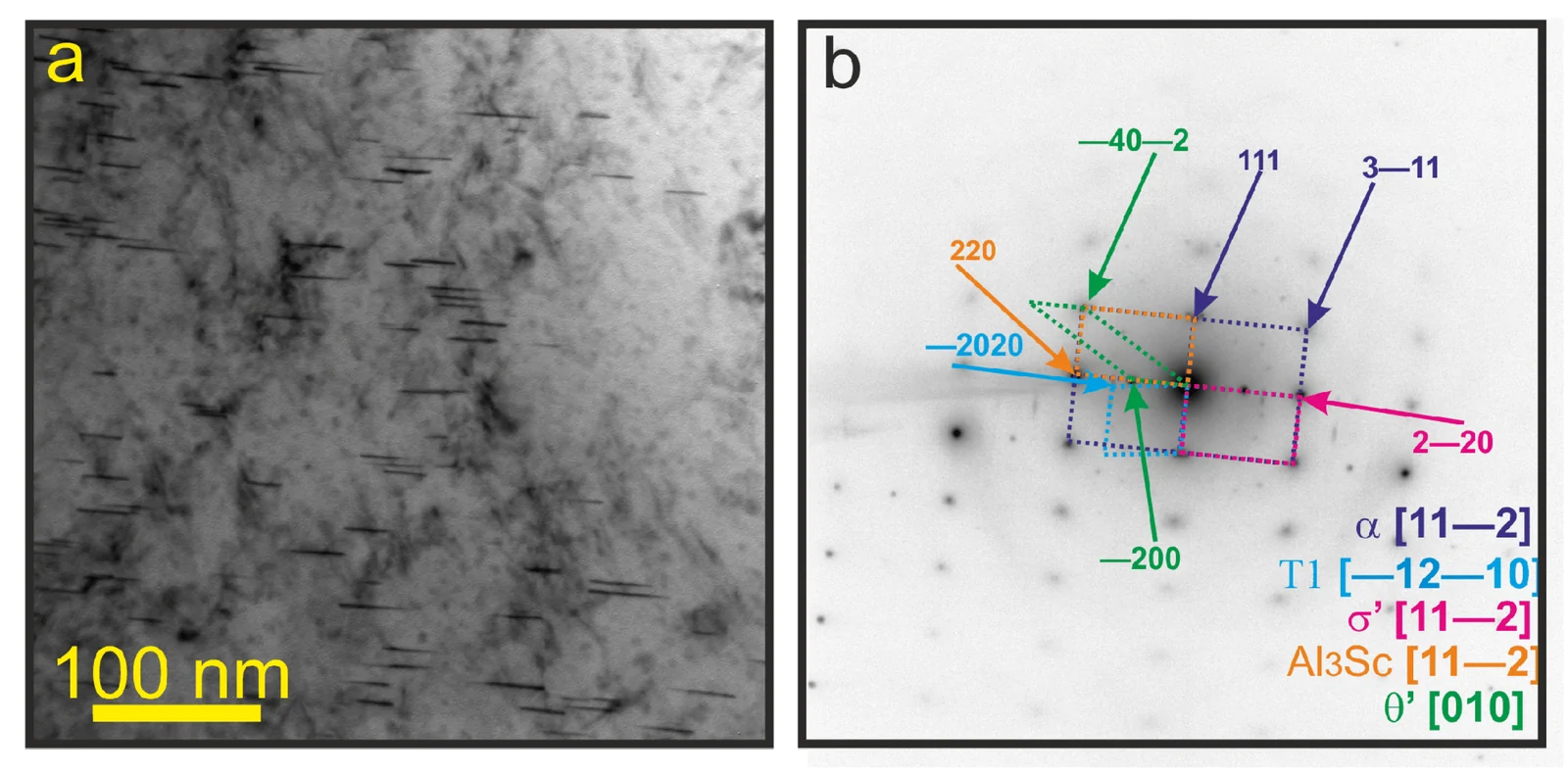
Fine structure (**a**) and SAED pattern (**b**) of an Al–Cu–Li alloy FSWed under air cooling.

Air-cooling in FSW on an Al–Cu–Li alloy creates conditions for rapid precipitation of reinforcement phase particles (Figure 4). According to identification using both SAED pattern and dark-field image, those are mainly T_1_-(Al_2_CuLi) and θ′-(Al_2_Cu) ones.

**Figure 5:**
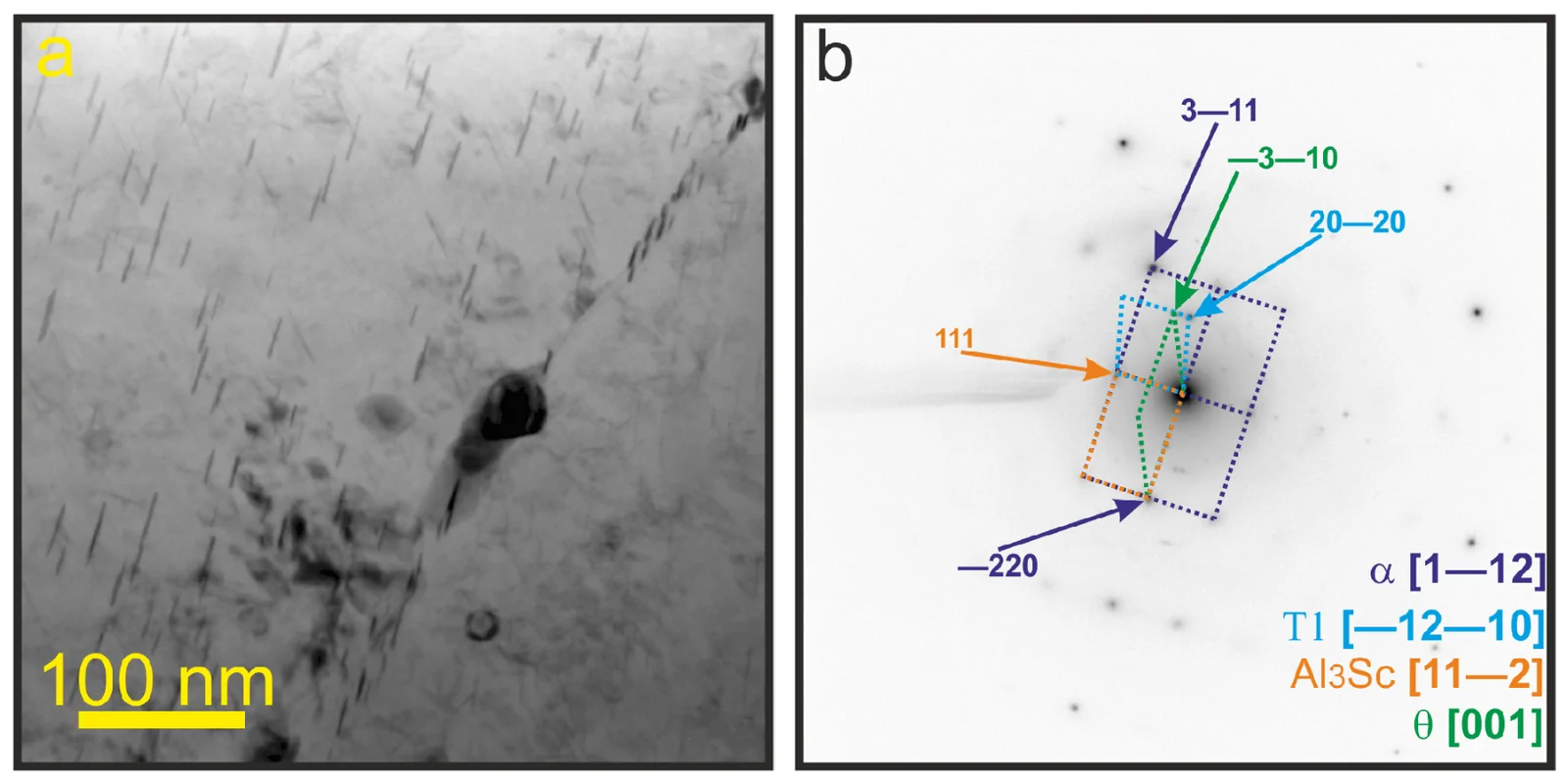
Fine structure (**a**) and SAED pattern (**b**) of an Al–Cu–Li alloy FSWed under liquid cooling.

The use of liquid cooling resulted in the precipitation of thinner and shorter T1-(Al_2_CuLi) and θ′-(Al_2_Cu) particles (Figure 5).

**Figure 6:**
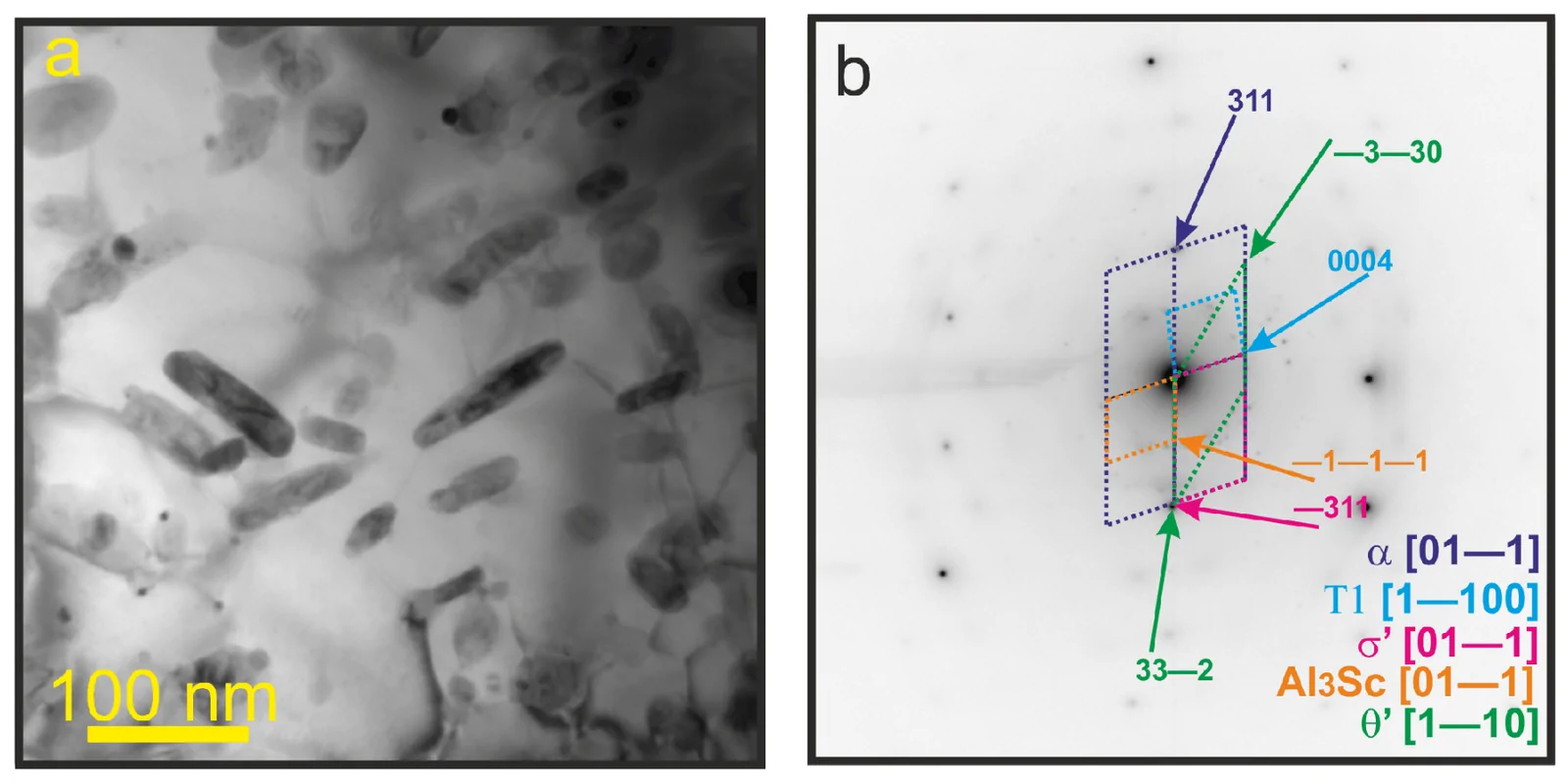
Fine structure (**a**) and SAED pattern (**b**) of an Al–Cu–Li alloy FSWed under liquid cooling with subsequent aging at 170 °C for 32 h.

Aging after water cooling leads to the coarsening of dispersed particles and an increase in their volume fraction (Figure 6). Some particles may lose coherence with the matrix, but the formation of new GPs helps to strengthen the alloy.

### 3.4. Fine SZ Structure in an FSWed Al–Zn–Cu–Mg (Ag, Zr, Sc) Alloy

**Figure 7:**
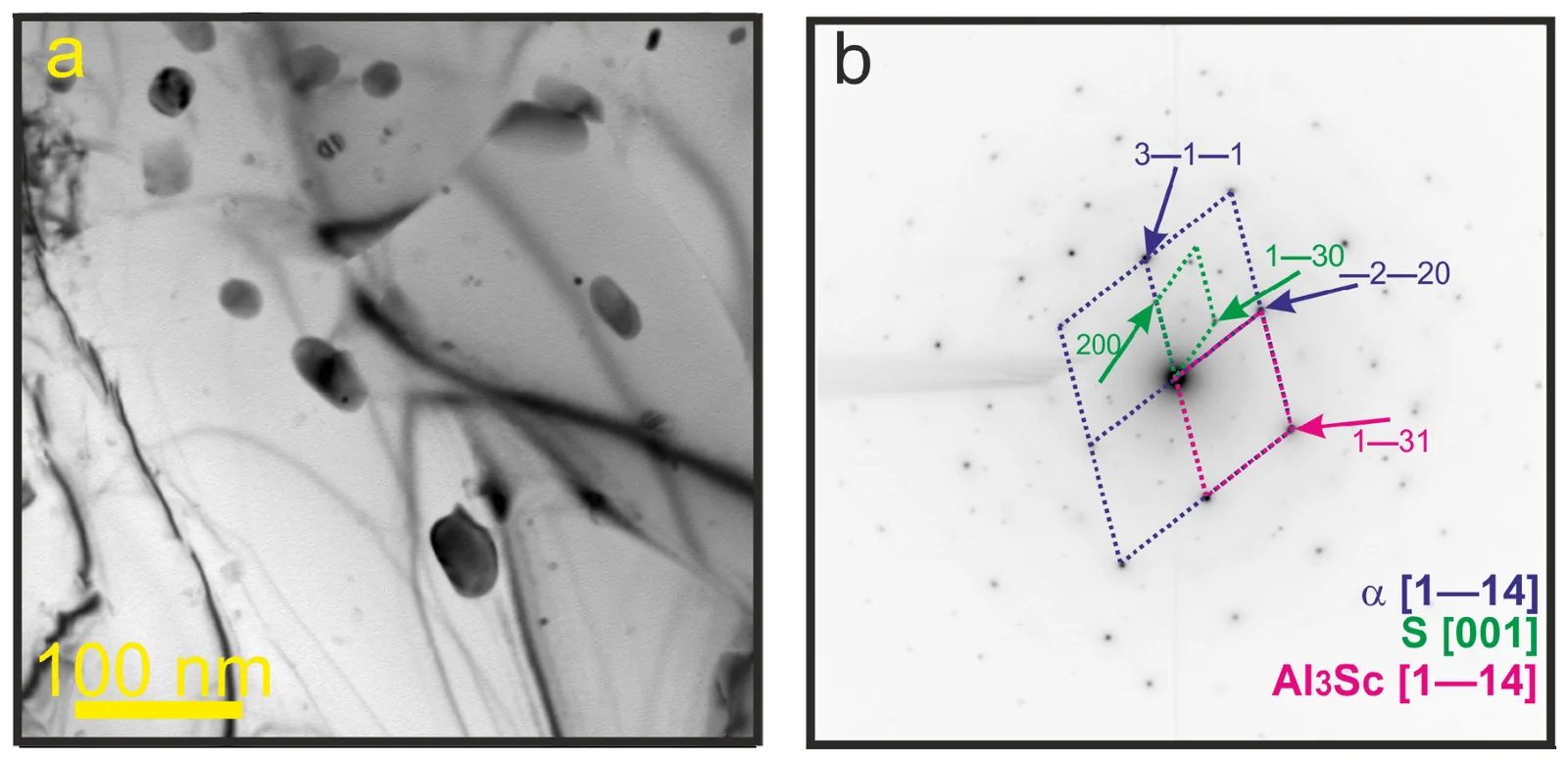
Fine structure (**a**) and SAED pattern (**b**) of an Al–Zn–Cu–Mg (Ag, Zr, Sc) alloy FSWed under air cooling.

The presented TEM images of structures obtained in the alloy stir zone formed in FSW with air cooling show the presence of both coarse and fine precipitates in the grains of the solid solution (Figure 7).

**Figure 8:**
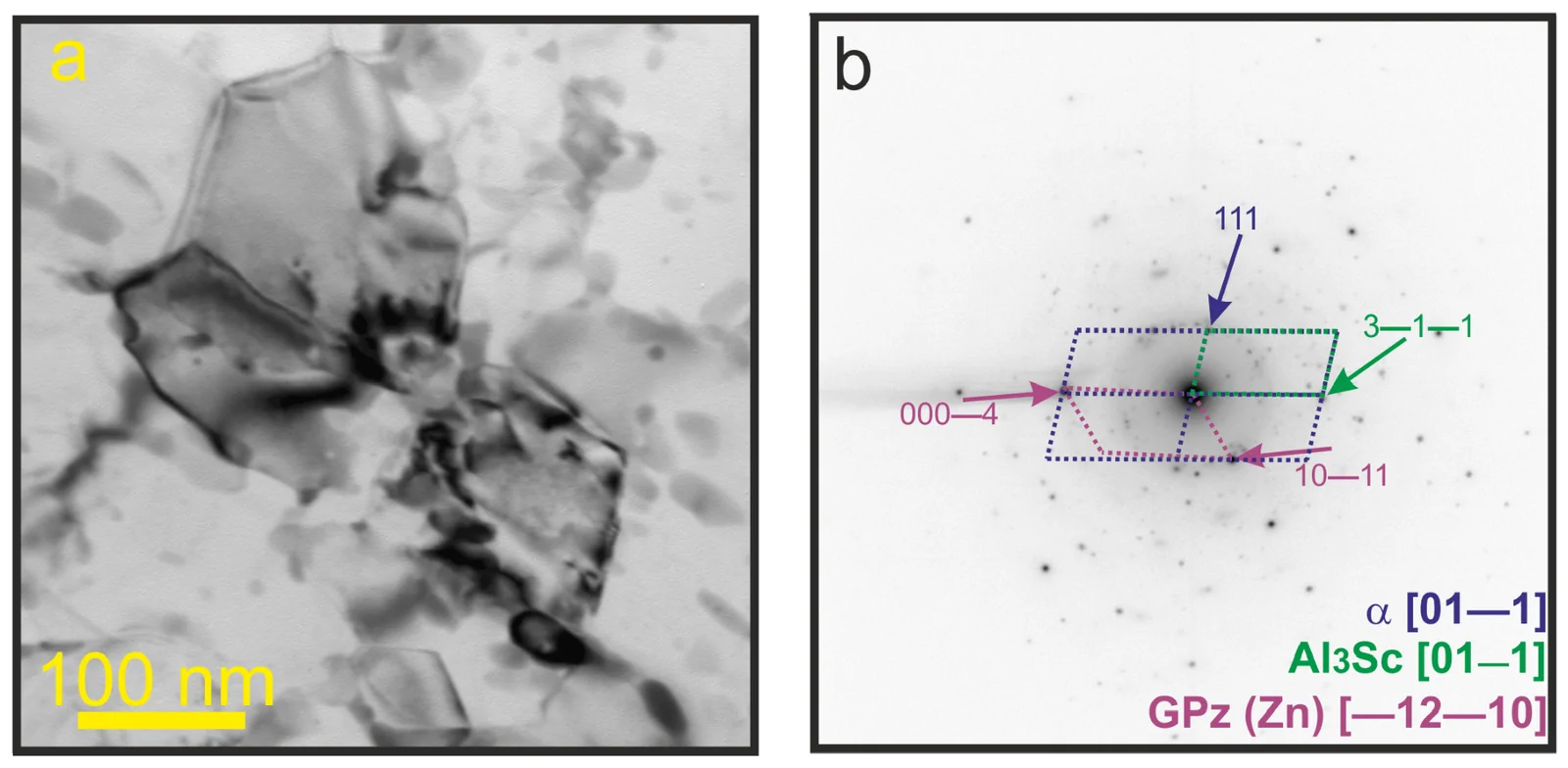
Fine structure (**a**) and SAED pattern (**b**) of an alloy FSWed under liquid cooling.

The dynamically recrystallized grains within the SZ of the liquid-cooled FSW joint exhibit a high density of fine, coherent Guinier–Preston (GP) zones. These nanoprecipitates are characterized by distinct strain-induced bend contours flanking their peripheries, which directly indicates the development of localized elastic strain fields caused by the structural lattice mismatch between the aluminum matrix and the coherent particles (Figure 8).

**Figure 9:**
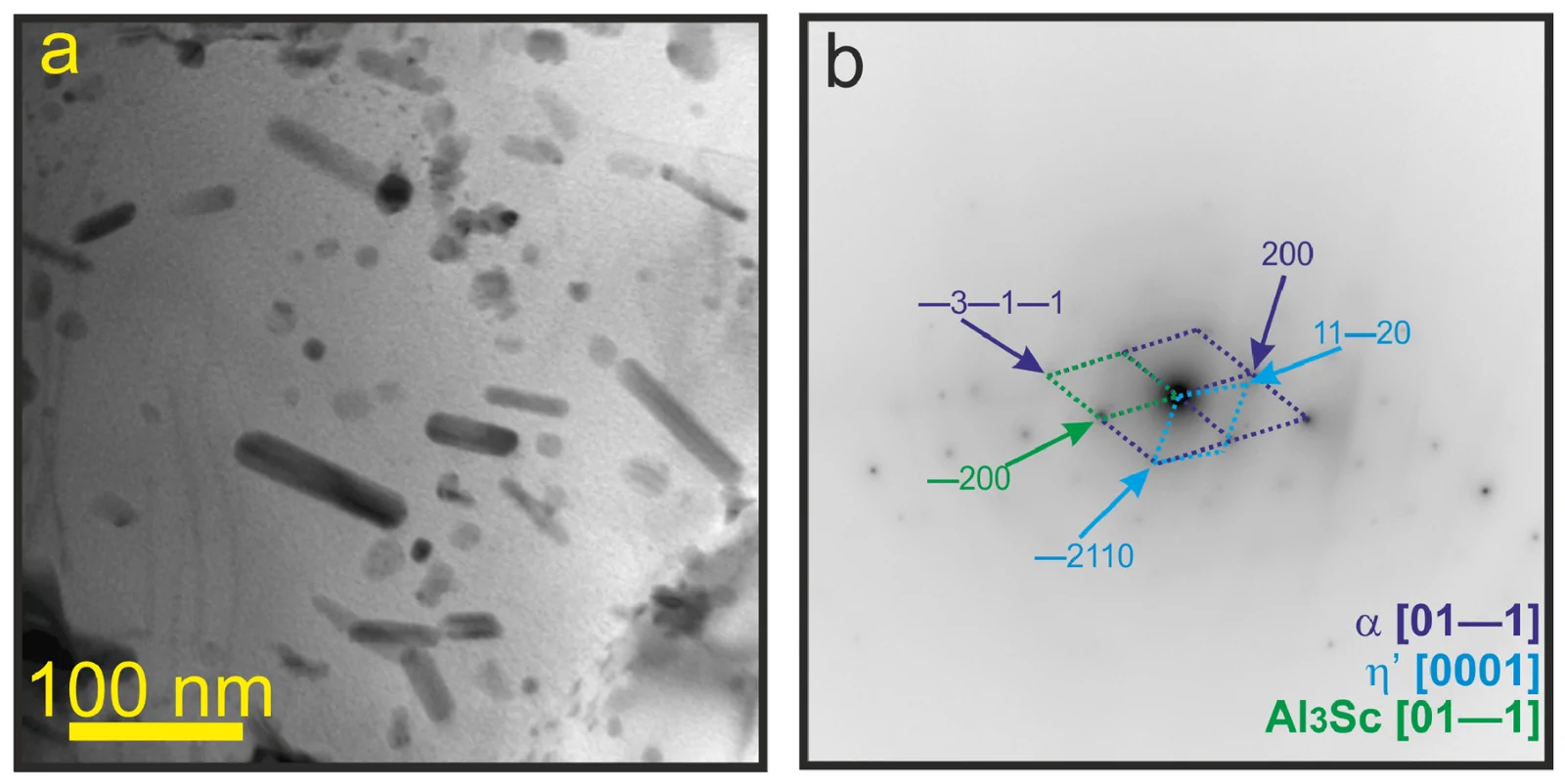
Fine structure (**a**) and SAED pattern (**b**) of an Al–Zn–Cu–Mg (Ag, Zr, Sc) alloy FSWed under liquid cooling with subsequent aging at 160 °C for 36 h.

Aging resulted in coarsening of the already existed GP zones as well as precipitation of numerous new and GP zones and fine particles (Figure 9).

### 3.5. Mechanical Properties of the Weld Metal Within the Stir Zones of Al–Cu–Li and Al–Zn–Cu–Mg (Ag, Zr, Sc) Alloys

**Figure 10:**
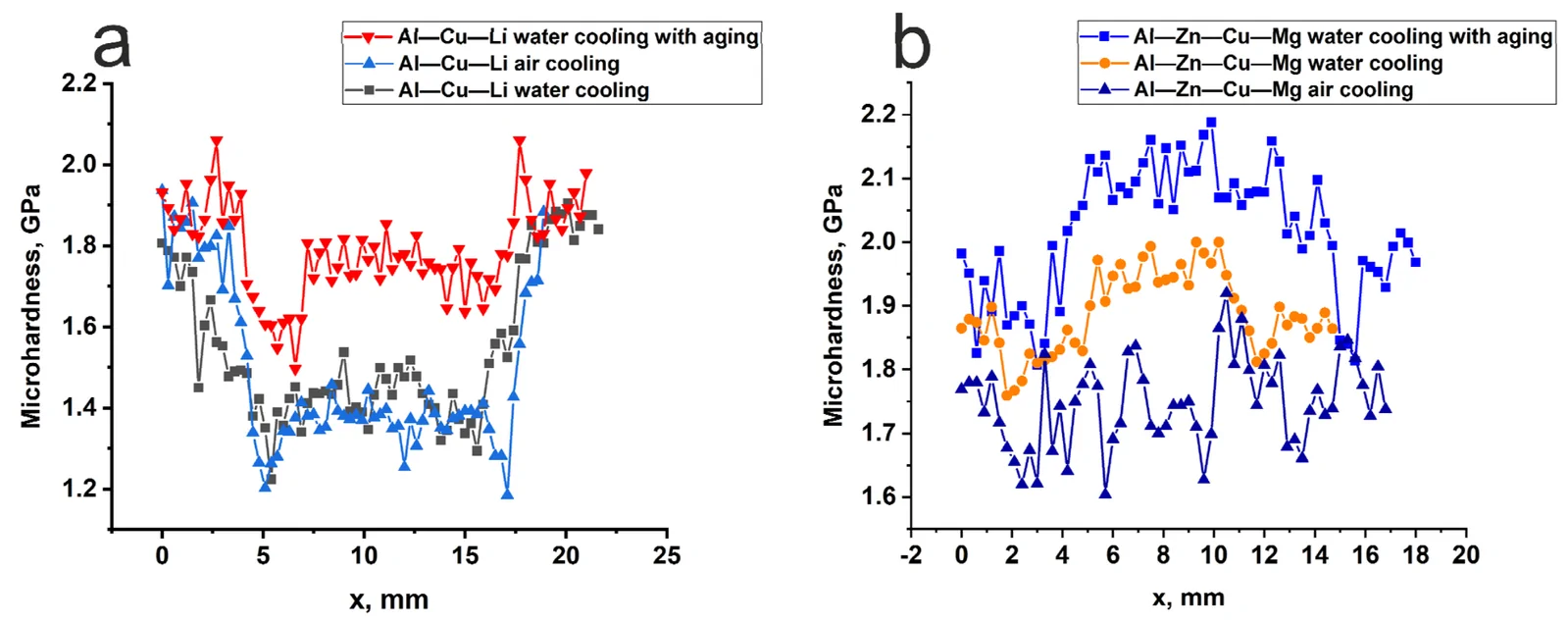
Microhardness number profiles in the cross-section of mixing zones in alloys (**a**) Al–Cu–Li and (**b**) Al–Zn–Cu–Mg (Ag, Zr, Sc) depending on cooling conditions in FSW.

The microhardness profiles across the weld joints indicate that for the Al–Cu–Li alloy, the SZ matrix subjected to post-weld artificial aging following forced water cooling exhibits the highest hardness response. Concurrently, in the initial as-welded condition, the water-cooled joint possesses a lower hardness compared to the counterpart processed under ambient air-cooling conditions (Figure 10a). Both profiles exhibit structurally “weakened” TMAZ regions, thereby manifesting a characteristic W-shaped distribution geometry. Despite the highly refined, fine-grained structures within the stir zone, the SZ microhardness levels in both the air- and liquid-cooled as-welded states remain substantially lower than that of the unaffected base metal. For the artificially aged FSW joints, however, this hardness deficit is significantly minimized, indicating that a high degree of hardness homogenization across the weld joint has been successfully achieved.

An inverse dependency regarding the impact of the cooling conditions is observed within the stir zone (SZ) of the Al–Zn–Cu–Mg alloy. Here, the microhardness in the as-welded state after water-cooled FSW is explicitly higher than that obtained under ambient air-cooling conditions (Figure 10b). Meanwhile, subsequent artificial aging yields the maximum hardness levels, demonstrating a post-weld precipitation hardening behavior that is conceptually consistent with the Al–Cu–Li system. Although the microhardness profiles in the 7xxx alloy series still retain softened zones within the TMAZ, the artificially aged SZ hardness levels are equal to or even surpass those of the initial base metal matrix.

**Figure 11:**
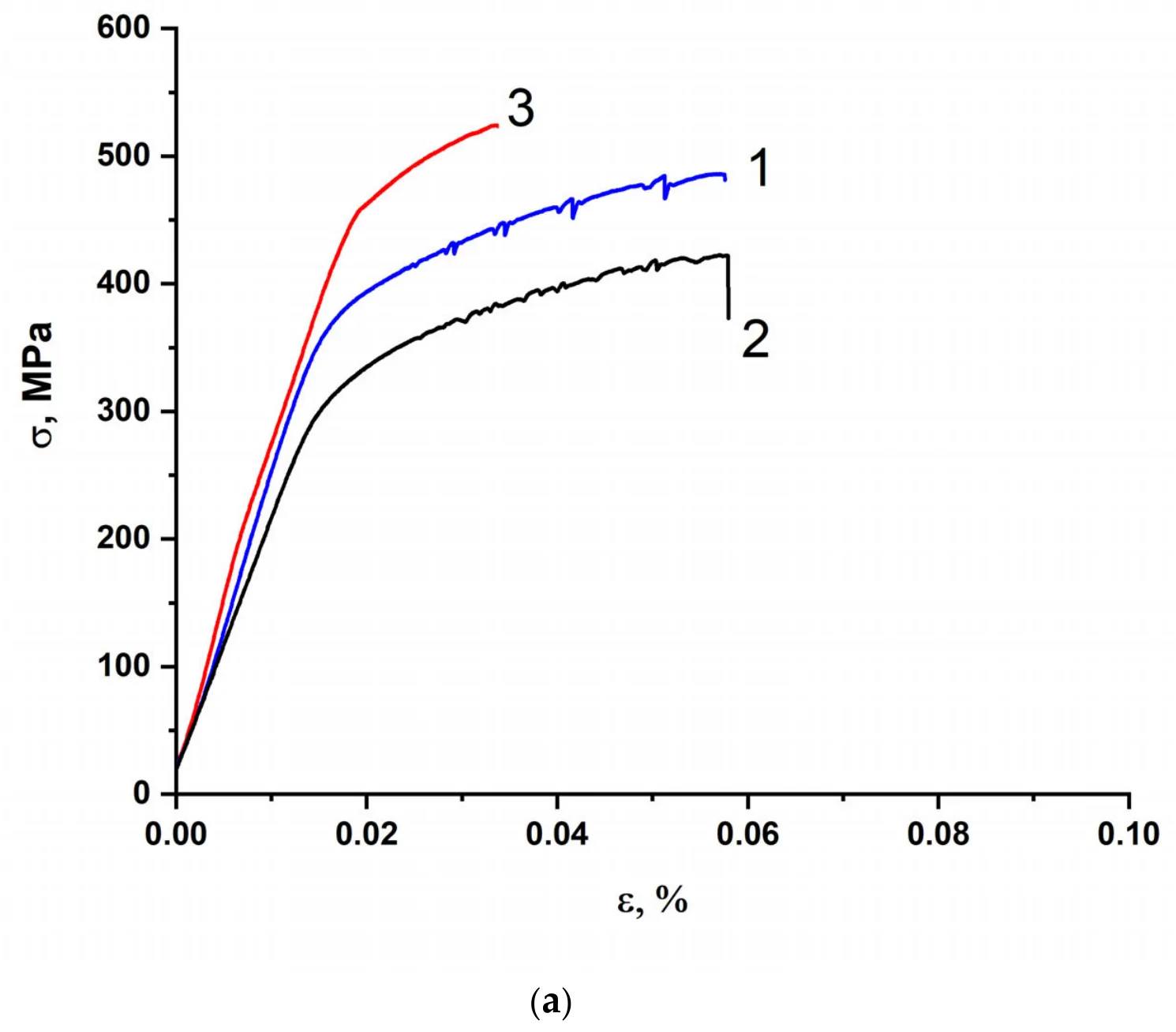

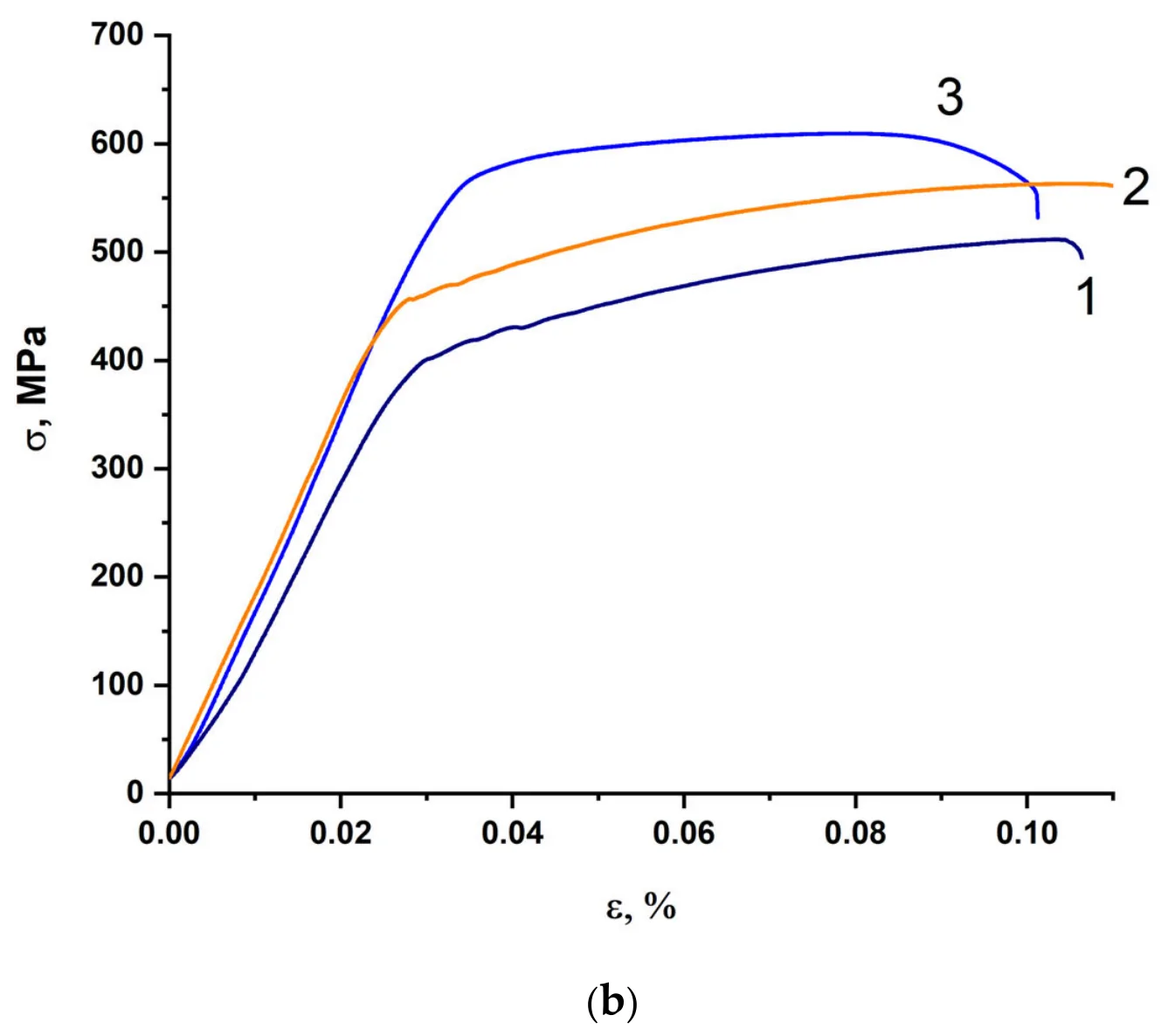
Engineering tensile stress–strain curves of the weld metal within the stir zones of (**a**) Al–Cu–Li and (**b**) Al–Zn–Cu–Mg (Ag, Zr, Sc) alloys depending on the FSW cooling conditions. 1—air cooling, 2—liquid cooling, 3—liquid cooling with subsequent aging.

The joint on the Al–Cu–Li alloy after liquid cooling and subsequent artificial aging exhibits the highest strength (Figure 11a). At a relative strain of approximately 0.02, the stress reaches about 450 MPa, and it climbs to 520–525 MPa toward the end of the test. The lowest deformation resistance was obtained for the liquid-cooled joint without post-weld artificial aging, where the stress at the end of the test is around 390 MPa (Table 2). This decrease in strength is consistent with the presence of local microhardness minima in the central region of the weld. Rapid cooling after welding creates a structural state that does not provide full precipitation hardening, meaning that subsequent aging is essential to unlock the potential of this state.

The tensile curve of the as-welded air-cooled specimen exhibits distinct, repetitive load drops (serrations). In the liquid-cooled specimen, these fluctuations are weaker, and for the post-weld artificially aged condition, they are virtually absent. This type of serrated yielding is a classic manifestation of discontinuous yielding, widely known as the Portevin–Le Chatelier (PLC) effect. The underlying mechanism is governed by dynamic strain aging (DSA), where interstitial or substitutional solute atoms (primarily highly diffusive copper and lithium) migrate toward dislocation cores during plastic deformation [57]. This localized segregation forms stable Cottrell atmospheres that temporarily pin the mobile dislocations. Consequently, an elevated critical stress must be applied to detach the dislocations from these solute atmospheres, leading to the observed macro-scale load fluctuations.

Crucially, the intensity and critical strain for the onset of the PLC effect are deeply sensitive to the local thermal history, ambient temperature, and effective strain rate. In the air-cooled joints, the slow cooling rate allows for an optimal density of deformation-induced vacancies and a high concentration of free solute atoms in the matrix, which accelerates interstitial diffusion kinetics. The presence of highly diffusive lithium atoms expands the critical temperature-strain rate window where DSA operates natively at room temperature [57]. Conversely, intensive liquid cooling suppresses rapid solute migration by inducing a high elastic lattice shear strain barrier and promoting massive vacancy annihilation, thereby weakening the serration intensity. Subsequent post-weld artificial aging completely eliminates the PLC effect because the free solute atoms (Cu and Li) are fully depleted from the solid solution to form stable, coherent, and semi-coherent strengthening nanoprecipitates (T_1_ and θ′). With the matrix effectively depleted of solute atoms, the thermodynamic conditions required to sustain the cyclic pinning-unpinning of dislocations via Cottrell atmospheres are no longer satisfied.

For the Ag/Zr/Sc-modified Al–Zn–Cu–Mg alloy, the joint subjected to forced liquid cooling followed by post-weld artificial aging exhibits the maximum ultimate tensile strength of approximately 610 MPa (Figure 11b, Table 2). Upon reaching a flow stress of about 560 MPa, the stress–strain curve continues to rise smoothly, transitioning into a localized necking and stress-drop stage only immediately prior to final fracture. The as-welded liquid-cooled specimen reaches a notable strength level of approximately 560 MPa; however, its strain hardening behavior develops at a lower rate throughout the plastic deformation regime (Table 2). The lowest tensile strength, around 510 MPa (Table 2), was obtained under conventional ambient air-cooling conditions. Consequently, accelerated liquid cooling inherently enhances the mechanical properties of the Al–Zn–Cu–Mg joints compared to air cooling, whereas the maximum strengthening potential of the modified matrix is unlocked only after subsequent artificial aging.

**Figure 12:**
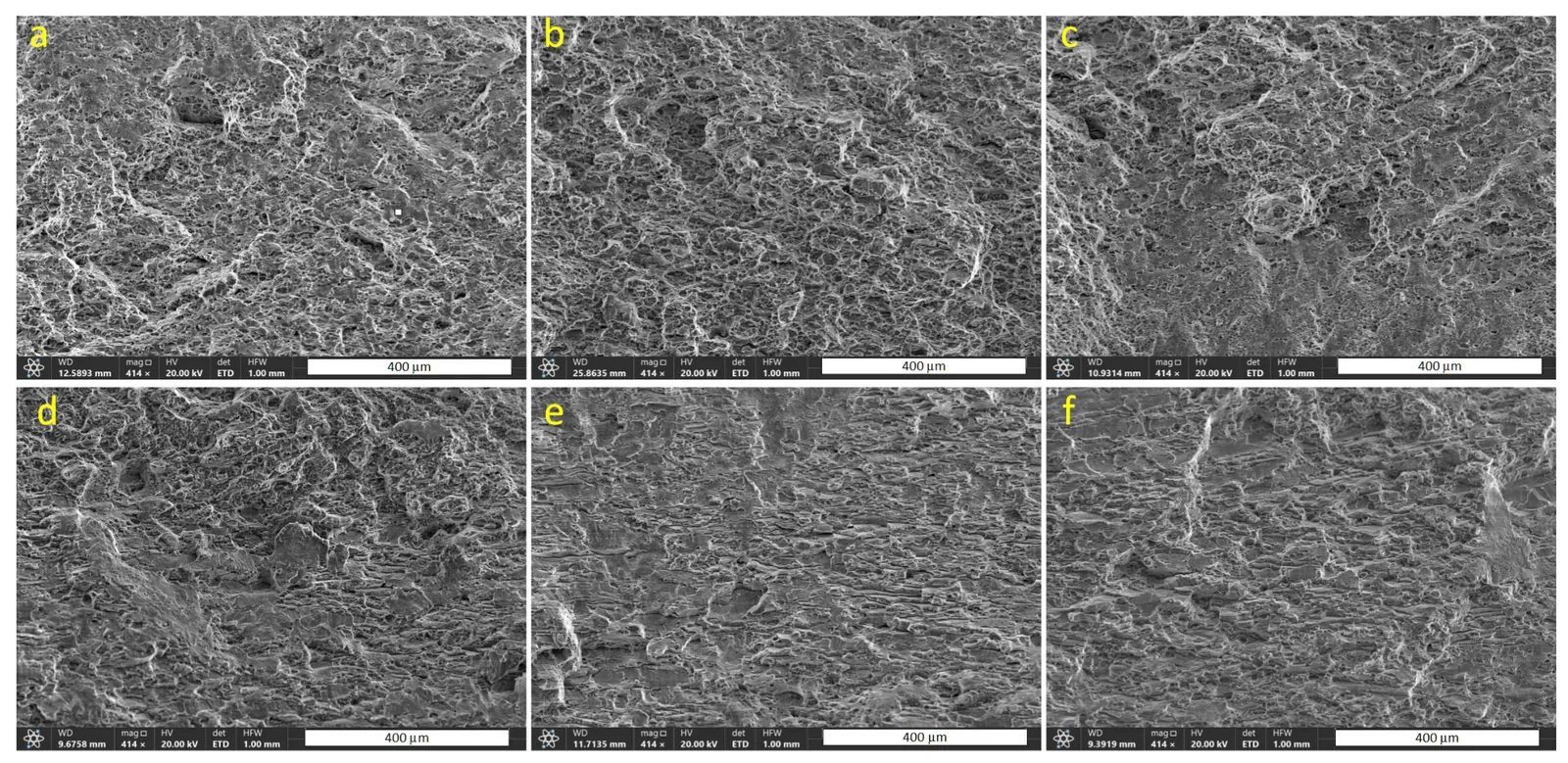
SEM SE images fracture surfaces in Al–Cu–Li (**a**–**c**) and Al–Zn–Cu–Mg (Ag, Zr, Sc) (**d**–**f**) alloy joints obtained using FSW with air- (**a**,**d**), liquid (**b**,**e**) and liquid cooling with aging (**c**,**f**).

Fracture surface morphologies obtained via scanning electron microscopy from the tensile-tested specimens (Figure 12) primarily reveal a characteristic distribution of microvoids and dimples, indicating a predominantly ductile fracture mechanism driven by microvoid coalescence. A notable exception is observed for the specimen shown in Figure 12c, where flat cleavage facets and distinct brittle fracture features are locally discernible across the fractographs.

## 4. Discussion

A relatively limited number of studies in the literature are dedicated to the FSW of advanced Al–Cu–Li system alloys. Specifically, a tensile strength of 435 MPa was reported for the 2060 Al–Cu–Li alloy joint [58]. Lukin et al. [59] achieved joint strength values ranging between 370 and 400 MPa for the V-1461 alloy, while a tensile strength on the order of 360–400 MPa was documented following the FSW of the 2198 alloy [60]. Furthermore, prior investigations have demonstrated that decreasing the tool rotational speed—thereby effectively mitigating the total thermal input—led to a noticeable increase in the tensile strength of FSWed 2195 alloy joints from 360 to 425 MPa [61]. Those findings strongly corroborate the core mechanism identified in the present study: reducing the peak temperature and shortening the thermal cycle within the stir zone directly contributes to a higher post-weld joint strength. For comparison, the ultimate tensile strength of the commercial V-1461 alloy typically ranges between 540 and 560 MPa, underlining that active thermal management is a critical factor in narrowing the gap between joint performance and base metal properties.

Consequently, based on the documented literature data, FSW joints in Al–Cu–Li system alloys consistently manifest a joint efficiency deficit (i.e., they lack mechanical homogeneity across the joint zones). This strength mismatch occurs because uncontrolled precipitation pathways and the heterogeneous dissolution of strengthening phases result in distinct localized mechanical properties across the weld. This joint efficiency deficit represents a critical limitation for structural reliability, as it can induce strain localization and accelerate premature failure during service life exploitation. Managing the precipitation kinetics within the characteristic FSW joint zones can be successfully achieved through active thermal management via accelerated cooling. In the present work, cooling regimes utilizing air and water jets were systematically evaluated. Additionally, post-weld artificial aging was performed on the specimens processed under forced water cooling.

The resulting mechanical characteristics indicate that in the initial as-welded state, ambient air cooling promotes a superior tensile response compared to water cooling alone. At the same time, the synergistic application of intensive water cooling and subsequent post-weld artificial aging yields the highest joint strength. This behavior is governed by the alteration of precipitation pathways under different cooling rates. While water cooling effectively suppresses dynamic precipitation during welding by preserving a maximum supersaturation state within the solid solution, subsequent artificial aging successfully triggers a massive, homogeneous co-precipitation of secondary phases. As established above, the dominant strengthening mechanism for this alloy system is driven by precipitation hardening via highly dispersed secondary phases, with the nanoscale T_1_ phase precipitates providing the most profound strengthening effect on the aluminum matrix.

During FSW, intense severe plastic deformation and localized material flow of the plasticized metal occur within the stir zone. This induces complete deformation-induced dissolution of the pre-existing dispersed strengthening precipitates within the aluminum matrix. Under the combined effects of extreme strain rates and frictional heating, these secondary phases subsequently undergo a series of transient reprecipitation and re-dissolution events, establishing a dynamic equilibrium between shear-induced dissolution and thermal precipitation kinetics. In the region trailing immediately behind the welding tool, precipitate nucleation and growth become the dominant phase transformation paths, occurring concurrently with the dynamic recrystallization and restructuring of the solid solution grains. These newly formed, highly dispersed secondary particles act as potent barriers to grain boundary migration via the Zener pinning mechanism, thereby effectively suppressing post-dynamic grain growth and contributing to significant grain refinement within the weld nugget. Depending on the thermo-mechanical conditions within the stir zone, the following structural scenarios may arise. Accelerated growth of the strengthening particles can lead to an overaging effect and the subsequent loss of their strengthening efficiency [62], which is characterized by a distinctive microhardness drop within the stir zone and thermo-mechanically affected zones [63]. The presence of such weakened zones cannot be remediated by post-weld aging. Conversely, when particle growth is suppressed, an underaging effect can be achieved, which can be readily eliminated through subsequent artificial aging. Therefore, achieving enhanced mechanical properties in heat-treatable aluminum alloy joints necessitates the implementation of forced cooling.

In addition to temperature and thermal dwell time, the precipitation kinetics of the strengthening phases in Al–Cu–Li alloys depend heavily on the local dislocation density, as dislocations act as preferential heterogeneous nucleation sites by reducing the activation energy barrier [64]. The occurrence of dynamic recrystallization (DRX) during FSW demonstrates that a specific density of these lattice defects is continuously generated, which inherently alters the subsequent phase precipitation pathways. Consequently, a complex, coupled mechanism governs how the localized cooling rate dictates phase stability, spatial distribution, and the resulting macroscopic strength of the joints.

The contrasting effects of the post-weld cooling rate on the as-welded tensile response of FSW joints in Al–Cu–Li versus AA7075 alloys, along with the complex multi-phase precipitation sequence of intermetallics in the Al–Cu–Li system, can be rigorously rationalized from the standpoint of Classical Nucleation Theory (CNT) and the Lifshitz–Slyozov–Wagner (LSW) kinetic model for diffusion-controlled coarsening. The mechanical properties of the joints after prolonged artificial aging (170 °C for 32 h for Al–Cu–Li and 160 °C for 36 h for Al–Zn–Cu–Mg) are fundamentally governed by a competitive kinetic trade-off between primary/secondary phase nucleation and aggressive Ostwald ripening localized within the dynamically recrystallized grains of the stir zone (SZ).

### 4.1. Microstructural Evolution and Mechanical Properties of the Weld Metal Within the Stir Zone After Air-Jet FSW

Cooling with a compressed air jet provides an intermediate (“controlled”) heat dissipation rate, which causes the weld zone to remain within the high-temperature diffusion interval for a longer duration. Within the stir zone (SZ) of the Al–Cu–Li alloy, this temperature range activates the efficient transport of lithium and copper atoms. The high dislocation density, which is generated by intense plastic deformation during FSW, can facilitate the heterogeneous nucleation of particles. Under these conditions, intensive dynamic precipitation of dispersed T_1_ (Al_2_CuLi) nanoplates occurs along the {111}_Al_ habit planes. Consequently, in the as-welded condition, the tensile strength of the air-cooled Al–Cu–Li specimen is noticeably higher (~450 MPa) than that of the water-cooled one (~390 MPa), which is also supported by the elevated microhardness level (~1.40–1.50 GPa) within the SZ.

In contrast, the Al–Zn–Cu–Mg (Ag, Zr, Sc) system alloy (AA7075) exhibits classical features of high quench sensitivity under the exact same conditions. During air cooling, the highly mobile zinc and magnesium atoms migrate avalanche-like from the grain interiors toward defect sinks, namely the dislocation walls and grain boundaries within the SZ. There, they heterogeneously precipitate as coarse, thermodynamically stable, yet incoherent particles of the η-MgZn_2_ phase. This process leads to an irreversible local depletion of the solid solution within the grain matrix. As a result, the tensile strength of the specimens after air-cooled FSW drops to its minimum values (~500 MPa), while the microhardness profile within the SZ demonstrates a pronounced decline accompanied by severe fluctuations in the range of 1.65–1.80 GPa.

### 4.2. Microstructural Evolution and Mechanical Properties of the Weld Metal Within the Stir Zone After Water-Cooled FSW

The application of intensive water cooling fundamentally alters the initial thermodynamic state of the stir zone. The ultra-high heat dissipation rate enables successful bypassing of the critical noses of high-temperature phase transformations on the continuous cooling transformation (CCT) diagrams. Water cooling promotes the retention of alloying elements dissolved within the aluminum lattice, thereby forming a supersaturated solid solution (SSSS). In the Al–Cu–Li alloy, the thermal lattice contraction caused by contact with water triggers a massive collapse and annihilation of excess thermal vacancies. Because lithium exhibits an anomalously high binding energy with vacancies (~0.25 eV) [65], the disappearance of free vacancies blocks the transport of Li atoms and renders the accommodation of elastic shear strains impossible, which are necessary for restructuring the FCC matrix lattice into the hexagonal structure of the T_1_-phase [66]. The nucleation of both the T_1_ phase and the semi-coherent θ′ (Al_2_Cu) phase is completely suppressed. Consequently, the weld metal within the stir zone remains in an underaged condition, exhibiting a minimum tensile strength of ~390 MPa and a microhardness drop down to ~1.35–1.40 GPa within the SZ.

In the AA7075 alloy, water cooling suppresses the growth and coalescence of the stable η-phase at the grain boundaries, allowing the Zn and Mg atoms to remain in the solid solution. However, unlike the Al–Cu–Li system, the Al–Zn–Mg–Cu system is highly prone to barrierless homogeneous clustering [67,68]. Within the recrystallized structure of the SZ, an avalanche-like natural aging process (Zn–Mg clustering) is initiated. The remaining deformation defects catalyze this process, promoting the formation of a high density of nanoscale Guinier–Preston (GP) zones. This gives rise to an anomalous, sharply localized central microhardness peak (up to 1.95–2.00 GPa) along the microhardness profile, which noticeably exceeds the hardness obtained after air-cooled FSW.

### 4.3. Phase Transformation and Coarsening Kinetics During Post-Weld Artificial Aging After Water-Cooled FSW

The application of subsequent artificial aging (post-weld heat treatment, PWHT) completely shifts the strength balance in favor of the water-cooled joints for both alloys. According to classical nucleation theory (CNT), the steady-state nucleation rate *I_s_* depends exponentially on the critical thermodynamic barrier (ΔG*) [69]:

(1) $$I_{s}=N_{0}\exp\left(-\frac{{∆G}^{*}+{∆G}_{m}}{k_{B}T}\right)$$

N_0_ is the atomic site density, *ΔG_m_* is the activation energy for atomic migration, k_B_ is the Boltzmann constant, and T is the absolute aging temperature. The thermodynamic barrier for the formation of a critical nucleus is defined as:

(2) $${∆G}^{*}=\frac{16\pi \gamma^{3}}{{3({∆G}_{\vartheta }-{∆G}_{s})}^{2}}$$

where γ is the specific interfacial energy, ΔG_s_ is the elastic strain energy of the lattice (induced by structural mismatch), and *ΔG_v_* is the volumetric chemical driving force.

When heating the water-cooled Al–Cu–Li specimen to 170 °C (with a 32 h dwell time), thermal energy activates the diffusion of vacancy–solute complexes. Since the matrix was retained in a clean supersaturated solid solution state, the chemical driving force ΔG_v_ is maximized, which lowers the barrier ΔG^*^ practically to zero and minimizes the critical nucleus radius (r^*^). This triggers a powerful homogeneous drive toward the co-precipitation of phases: ultra-thin T_1_ nanoplates along the {111}_Al_ planes and semi-coherent θ′-phase plates along the {100}_Al_ planes. Their mutual interleaving forms an ultra-dense, reinforcing interpenetrating structure within the SZ grains, raising the ultimate tensile strength to a peak value of ~525 MPa and the microhardness to ~1.80 GPa. Similarly, aging the water-cooled AA7075 alloy at 160 °C for 36 h transforms the GP zones into a dense, homogeneous system of metastable η′-phase nano-precipitates due to the massive *ΔG_v_* driving force, resulting in an extreme leap in tensile strength to >600 MPa and microhardness to ~2.20 GPa.

In contrast, the air-cooled specimens enter the aging stage with an already depleted matrix and relatively coarse pre-existing nuclei (*r*_0_ > 0) bypassing the nucleation stage. Upon heating, they immediately transition into the kinetic regime of diffusion-controlled Ostwald ripening, described by the cubic power law of the LSW theory [70,71,72,73]:

(3) $$r^{3}\left(t\right)-r_{0}^{3}=K_{\mathrm{LSW}}\cdot t$$

where the particle coarsening rate constant *K_LSW_* is expressed as:

(4) $$K_{\mathrm{LSW}}=\frac{8\gamma V_{m}^{2}DC_{\infty }}{9\mathrm{RT}}$$

where *V_m_* is the molar volume of the precipitate, *D* is the diffusion coefficient of the solute element, and *C_∞_* is the equilibrium solubility at a flat interface. According to the Gibbs–Thomson effect, the local solute concentration (*C_r_*) near the curved surface of a particle with a radius (*r*) is given by:

(5) $$C_{r}=C_{\infty }\exp\left(\frac{2\gamma V_{m}}{\mathrm{rRT}}\right)$$

The concentration gradient between fine and coarse particles forces the solute elements to migrate toward the pre-existing coarse precipitates η particles in AA7075 or coarse T_1_ in (Al–Cu–Li). Since the interfacial energy of the θ′-phase is significantly higher than that of the T_1_ (γ_θ′_ > γ_T1_) [74] its coarsening rate constant *K_LSW_* in the Al–Cu–Li alloy approaches higher values, causing complete degradation and dissolution of the θ′ nuclei. The chemical driving force of the matrix drops to zero (Δ*G_v_* → 0) completely blocking the possibility of secondary nucleation within the grain interiors. As a result, after 32 and 36 h of aging, the weld metals within the stir zones are characterized by an overaged structure containing coarsened particles with large interparticle spacing. Dislocations easily bypass these particles via the Orowan mechanism, which accounts for the low strength characteristics and a modest response to PWHT compared to the water-cooled joints.

## 5. Conclusions

Based on the comprehensive comparative investigation of FSW joints in high-strength Al–Cu–Li and modified Al–Zn–Cu–Mg alloys under variant cooling conditions, the following main conclusions can be drawn:

- A fundamental divergence in the microstructural stabilization mechanisms was established for the first time under altered thermal cycles. While both alloys benefit from forced water cooling via suppressed dynamic recrystallization grain growth in the stir zone (SZ), their underlying phase stability mechanisms differ fundamentally.
- In the Al–Cu–Li alloy, the post-weld hardening response is governed by the thermal dissolution kinetics of the strengthening phases. It was uniquely demonstrated that accelerated water cooling partially prevents the complete dissolution of the metastable T_1_-Al_2_CuLi phase, directly driving a higher post-weld joint recovery.
- The Ag-, Zr-, and Sc-modified Al–Zn–Cu–Mg alloy exhibited unprecedented structural stability due to a powerful Zener pinning effect exerted by thermally stable Al_3_(Sc, Zr) nanoprecipitates. This mechanism successfully maintains an ultra-fine grain structure (≤1 µm) even under ambient air conditions and completely suppresses grain boundary shearing during rapid cooling.
- By optimizing the cooling rate via forced water cooling, a remarkable joint efficiency exceeding 85% was achieved for the modified Al–Zn–Cu–Mg alloy. This optimization successfully shifts the typical failure location away from the heat-affected zone (HAZ) and toward the thermomechanically affected zone (TMAZ) boundary, offering a novel pathway for structural optimization in high-strength aluminum FSW joints.

## Figures and Tables

**Table 1:** FSW Parameters.

Sample	Cooling	V, mm/min	n, rpm	Inclination, °
Al–Zn–Cu–Mg	Air	900	900	2.5
Al–Zn–Cu–Mg	Water			
Al–Cu–Li	Air			
Al–Cu–Li	Water			

**Table 2:** Mechanical properties of alloy FSW joints.

Sample and Cooling	σ, MPa	σ_0.2_, MPa	Strain, %
Al–Zn–Cu–Mg air	510	400	10.6
Al–Zn–Cu–Mg water	560	470	11
Al–Zn–Cu–Mg water with aging	610	560	10.1
Al–Cu–Li air	450	470	3.75
Al–Cu–Li water	390	310	3.4
Al–Cu–Li water with aging	520	370	3.75

## Data Availability

The original contributions presented in this study are included in the article. Further inquiries can be directed to the corresponding author.

## Abbreviations

The following abbreviations are used in this manuscript:

FSW	Friction Stir Welding
SZ	Stir Zone
TMAZ	Thermomechanically Affected Zone
HAZ	Heat Affected Zone
BM	Base Metal
GP	Guinier–Preston Zone
CNT	Classical Nucleation Theory
LSW	Lifshitz–Slyozov–Wagner
